# Analysis of the docking property of host variants of hACE2 for SARS-CoV-2 in a large cohort

**DOI:** 10.1371/journal.pcbi.1009834

**Published:** 2022-07-11

**Authors:** Hyojung Paik, Jimin Kim, Sangjae Seo

**Affiliations:** 1 Division of Supercomputing, Center for supercomputing application and research, Korea Institute of Science and Technology Information (KISTI), Daejeon, South Korea; 2 Department of Data and HPC science, University of Science and Technology (UST), Daejeon, South Korea; University of Maryland School of Pharmacy, UNITED STATES

## Abstract

The recent novel coronavirus disease (COVID-19) outbreak, caused by severe acute respiratory syndrome coronavirus 2 (SARS-CoV-2), is threatening global health. However, an understanding of the interaction of SARS-CoV-2 with human cells, including the physical docking property influenced by the host’s genetic diversity, is still lacking. Here, based on germline variants in the UK Biobank covering 502,543 individuals, we revealed the molecular interactions between human angiotensin-converting enzyme 2 (hACE2), which is the representative receptor for SARS-CoV-2 entry, and COVID-19 infection. We identified six nonsense and missense variants of hACE2 from 2585 subjects in the UK Biobank covering 500000 individuals. Using our molecular dynamics simulations, three hACE2 variants from 2585 individuals we selected showed higher levels of binding free energy for docking in the range of 1.44–3.69 kcal/mol. Although there are diverse contributors to SARS-CoV-2 infections, including the mobility of individuals, we analyzed the diagnosis records of individuals with these three variants of hACE2. Our molecular dynamics simulations combined with population-based genomic data provided an atomistic understanding of the interaction between hACE2 and the spike protein of SARS-CoV-2.

## Author summary

To understand diverse infection rate of novel coronavirus disease (COVID-19) depending on individuals, it is of great importance to elucidate the interaction between severe acute respiratory syndrome coronavirus 2 (SARS-CoV-2) and human cell. In this study, we conducted computational mutagenesis study to understand the relationship between variants of human angiotensin converting enzyme 2 (hACE2), which is the major human cell receptor for SARS-CoV-2 entry, and COVID-19 infection. Massive data analysis of UK biobank database allowed us to find substantial evidences of altered COVID-19 infection by hACE variants. In addition, molecular dynamics simulation provided us atomistic understanding of interaction between hACE2 and spike protein of SARS-CoV-2.

## Introduction

A novel coronavirus (SARS-CoV-2) causing a respiratory illness was first reported in December 2019 in China and rapidly spread worldwide [1,2]. The pandemic due to SARS-CoV-2, called the COVID-19 pandemic by the WHO, is still ongoing. As of May 24, 2022, over 500 million cases have been reported, with over 6 million deaths due to COVID-19. The unprecedented rapid development of vaccines effectively reduced the number of infections, but the appearance of variants of SARS-CoV-2, including Delta and Omicron, which are variants of concern, makes it difficult to end the current pandemic [3–9]. Since most vaccines and therapeutic drug developments target the spike (S) protein of SARS-CoV-2, it is of great importance to understand its interaction with human cells.

The S protein of SARS-CoV-2 consists of the S1 and S2 subunits, which are responsible for receptor recognition and membrane fusion, respectively. Viral entry is initiated by recognition and binding to host surface cellular receptors [10–12]. The S proteins of different coronaviruses bind to different cellular receptors. HCoV-229E interacts with human aminopeptidase N (hAPN) [13], and MERS-CoV binds to human dipeptidyl peptidase 4 (hDPP4 or hCD26) [14,15]. SARS-CoV and SARS-CoV-2 utilize human angiotensin converting enzyme 2 (hACE2) as a receptor for cell entry [11,12]. Upon binding to hACE2, the S protein undergoes cleavage and conformational changes facilitated by host proteases. S1 can be further divided into an N-terminal domain (NTD) and a C-terminal domain (CTD). SARS-CoV and MERS-CoV utilize the S1 CTD, called the receptor binding domain (RBD), to recognize the receptor.

Variants of SARS-CoV-2 include mutations in the S protein. For example, the Delta variant, which is a major variant, contains 13 mutations, including D614G, T478K, L452R and P681R, which play an important role in recognition of the immune system and transmissibility [8,16–18]. In vitro and in silico mutagenesis studies of the S protein have shown that single-nucleotide polymorphisms are relevant to COVID-19 pathogenesis and transmission [9,17]. Likewise, the mutation of hACE2 also affects viral infection. Deep mutagenesis studies have found evidence of which binding affinity varies due to variations in hACE2 and have found key residues responsible for binding affinity changes, such as the asparagine 90-glycosylation motif [19]. Fei [20] and Hadi-Alijanvand [21] reported the effect of hACE2 mutations that affected its binding to SARS-CoV-2 based on an overall compendium of human hosts’ diversity. Although most random mutations are deleterious, some mutations are inherited by natural selections, resulting in genetic diversity of the population. Thus, due to the host dependency of pathogens, including SARS-CoV-2, understanding the genetic diversity of human hosts, such as germline variants of hACE2, is indispensable to shed light on the evolution of SARS-CoV-2 and the resistance of human hosts. Yanan et al. presented the genetic diversity of hACE2 across populations and suggested that a diverse genetic basis might affect hACE2 functions among populations [22]. Therefore, an understanding of the relationship between SARS-CoV-2 binding and natural genetic diversity on a nationwide scale is still lacking.

Moving forward, we investigated the impact of host genetic diversity on the entry of SARS-CoV-2. We performed both genetic data analyses covering 200,643 individuals represented in Whole Exome Sequencing (WES) data of the UK Biobank, which allowed us to detect rare and common variants in the population. In addition, we conducted molecular dynamics (MD) simulations to scrutinize the relationship between genetic variants of hACE2 and COVID-19 infection. The purpose of this study is twofold: 1) to reveal the effect of hACE2 germline variants on COVID-19 infection based on population data and 2) to understand the molecular mechanisms of hACE2 variants on the binding affinity of SARS-CoV-2.

## Results

### Identification of genetic variants of hACE2 in a population

To investigate the impact of hACE2 variation on its interaction with the S protein of SARS-CoV-2, we analyzed UK Biobank data covering over 500,000 individuals. Out of 502,543 of those participants, we utilized the WES data of 200,643 individuals. **Table 1** shows the statistical summary of the participants in the UK Biobank and the individuals whose WES data were analyzed. Analyzed data included mean age, sex, degree of obesity, Townsend deprivation index scores, physical status, and background (i.e., social environment) of the UK Biobank participants. The Townsend deprivation index is a measure of material deprivation within a population [23]. Lower Townsend deprivation index scores indicate less deprivation, such as stable employment and car ownership. The 502,591 individuals in the UK Biobank dataset are mostly middle-aged (mean age 56.53 ± 8.09) and middle class. These trends were also observed in the analyzed individuals who had WES data.

**Table 1:** Sociodemographic characteristics of UK Biobank participants*.

Features	No. of features (Total, n = 502,543)	No. of features (Those with WES data, n = 200,643)
Demographic features Mean age Sex Health-related features Height (cm) Weight (kg) Hospital admissions Socioeconomic features Townsend deprivation score	56.53 (±8.09) Male: 229,138 Female: 273,405 170.1 (±9.44) 76.40 (±15.94) 2,577,360 (Mean 6.52 per individual) -1.29 (±3.09)	56.46 (±8.10) Male: 90,153 Female: 110,477 170.0 (±9.38) 77.92 (±15.85) 984,218 (Mean 6.22 per individual) -1.33 (±3.05)

**Fig 1:**
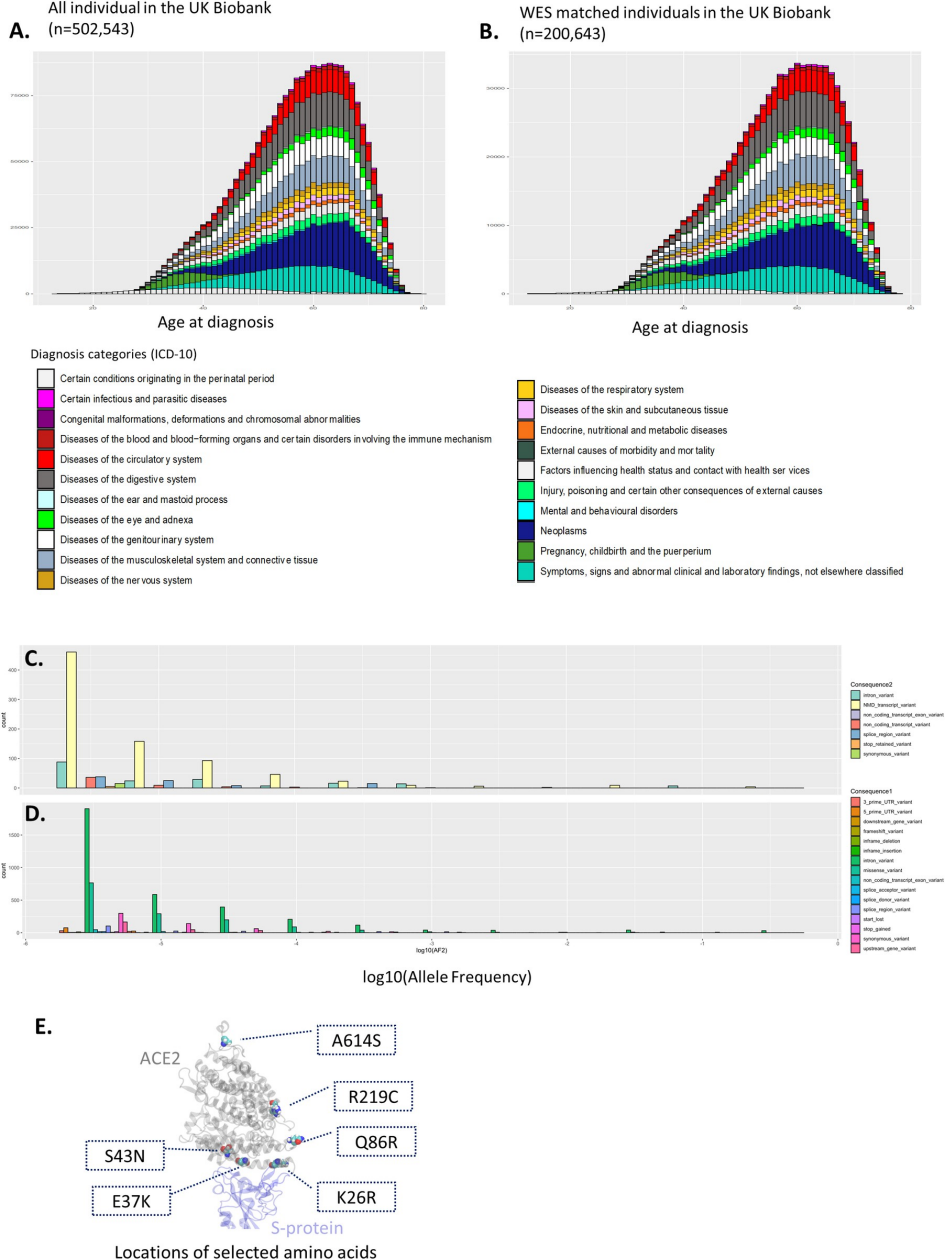
Analysis of UK Biobank participants and selected amino acids for mutation study.

Using assigned diagnosis codes in inpatient cases, we also evaluated health-related outcomes between all individuals in the UK Biobank and the WES data subset of the UK Biobank. A total of 2,577,360 primary diagnoses from the inpatient records were analyzed. Out of those diagnosis records, 984,218 cases were from the hospital admission data of 200,643 WES data-matched individuals. Based on the medical data of the UK Biobank, **Fig 1A** depicts known diagnosis trends by age and sex and health-related outcomes in general. For example, younger age at pregnancy (<40 years, green bars), increased number of diagnoses of the circulatory system (>50 years, red bars) and neoplasms (>40 years, dark navy bars) in older groups are obvious. Likewise, a subset of individuals who had matched WES data showed identical diagnostic trends (**Fig 1B**). Therefore, we felt confident that the UK Biobank represents adult populations in the UK. Then, we sought to verify the genetic diversity of hACE2 coding regions to address the functional impact on SARS-CoV-2 entry in the UK population.

The Ensembl Variant Effect Predictor (VEP) [24] assessed the functional impact of given genetic variants of the hACE2 gene in the included 200,643 individuals. With rule-based approaches, the VEP presents the consequences of each genetic variant, including synonymous, nonsense-mediated decay (NMD), and nonsynonymous variants (i.e., missense variants). Then, via the VEP, the degree of functional impact by the consequence of transcript variants is categorized into four groups: ‘HIGH (truncation of protein)’, ‘MODERATE (nondisruption and altered effect of protein)’, ‘MODIFIER (variants of noncoding region)’, and ‘LOW (unlikely to change the protein)’. Within the coding region of hACE2, 701 germline variants were detected. Out of those 701 variants, 10 were categorized as high-impact variants, including frameshift variants, stop-gained variants, NMD variants, and NMD of splice sites, whereas 96 variants showed a low impact of proteins, such as synonymous variants and intron variants. Details are presented in **S1 File**. In addition, we also analyzed the minor allele frequency (AF) of those identified variants. As depicted in **Fig 1C and 1D**, the majority of genetic variants with low functional impacts, such as synonymous variants, showed a higher allele frequency (mean AF = 0.00219±0.016), while variants with high functional impact, including missense variants and stop-gain variants, were rarely observed (mean AF = 5.105E-06±7.215E-06). Since common variants (i.e., high AF) presented a relatively moderate functional impact in general, the distribution of AF in hACE2 shows favorable consistency with a previous trend [25].

The variants showing high impact mostly contain stop codons in the hACE2 gene, indicating truncation of the hACE2 protein as a docking partner of SARS-CoV-2. Because we were interested in molecular interactions between hACE2 and SARS-CoV-2, we analyzed 181 variants with ‘moderate’ impact, including in-frame deletions (insertions), missense variants, and splice region variants (mean AF = 0.000214±0.021). Out of those 181 hACE2 variants, we selected six variants based on the locations of corresponding amino acids to investigate the effect of atomic contacts. **Fig 1E** depicts the locations of selected amino acids. Amino acid K26 is in close proximity to the RBD of the S protein, and R219 is located at the secondary shell to the interface. Q86 and A614 are separate from the interface.

Based on our analysis of genetic variants of hACE2 in the UK population, we identified six variants (n = 2585 individuals, mean AF = 0.001367667± 0.0024) as promising candidates for counterparts affecting SARS-CoV-2 entry. All those identified variants are germline variants. In further analysis, we addressed the molecular interactions between SARS-CoV-2 and each variant in the human host partner hACE2 based on alchemical free energy analysis.

### Alchemical free energy analysis by hACE2 variants

We first estimated the free energy changes to investigate the molecular basis for the effect of hACE2 variation on SARS-CoV-2 binding. **Table 2** lists the selected variants and corresponding calculated ΔΔG values. Except for the mutation of S43N, the selected mutations yielded significant ΔΔG values, which can substantially affect binding affinity. The variants K26R, E37K and A614S showed positive ΔΔG values of 3.10, 3.69 and 1.44 kcal/mol, respectively, which implied weaker binding affinity in the variants. On the other hand, the variants S43N, Q86R and R219C showed negative ΔΔG values of -0.50, -1.32 and -1.85 kcal/mol, respectively, indicating stronger binding affinity in the variants. The residues at the contact sites (K26R and E37K) showed larger ΔΔG value changes than residues farther from the interface (R219C and A614S). This result implies that the changes in physicochemical characteristics of contact residues readily affect the binding affinity between *hACE2* and SARS-CoV-2. To examine susceptibility to SARS-CoV-2 of the germline variants of hACE2, we examined the retrieved health care records of the UK Biobank, including COVID-19 diagnostic test results.

**Table 2:** Calculated free energy changes due to germline variants.

Variant	ΔΔG (kcal/mol)	No. of subjects (no. of homozygous of minor allele)
K26R	3.10	2074 (580)
E37K	3.69	8 (0)
S43N	-0.50	9 (0)
Q86R	-1.32	23 (0)
R219C	-1.85	341 (78)
A614S	1.44	130 (2)

### Analysis of COVID-19 diagnostic test results by genetic variants of hACE2

Fifty-three percent of individuals (106,600) out of 200,643 with UK Biobank WES data received COVID-19 testing for the diagnostic confirmation of SARS-CoV-2 infections. Out of 243,309 evaluations, there were 17,872 cases (positive ratio of 16.7%). We evaluate the difference in the positivity rate of COVID-19 tests by the germline variants of those selected variants of hACE2 in **Table 2**. To examine the contribution of the variants of *hACE2*, we selected individuals from the major allele group (i.e., wild-type *hACE2*), which had similar distributions regarding age, sex, and body mass index (BMI) as the minor allele group (i.e., *hACE2* variant subjects) using propensity score matching analysis. [26] (refer to the Method section for details). Therefore, selected individuals from the minor and major allele groups had similar distributions of age, sex, and degree of obesity (p value of t test > 0.05), indicating that the confounding effect of these characteristics was negligible. Considering propensity score matching, we selected individuals from major allele groups as 100-fold of minor allele groups.

**Table 3** lists the selected variants and corresponding COVID-19 test results. Intriguingly, the comparison of the positivity rate between the major allele (control group, wild-type hACE2) and minor allele (case group, hACE2 variant) groups indicated that single-nucleotide substitution was associated with SARS-CoV-2 infection in all cases. The residues K26R and R219C exhibited higher positivity rates, and residues A614S and Q86R exhibited higher positivity rates with mutated amino acids. However, the odds ratio (OR) of the K26R variant was 0.93, whereas the OR of R219C was 1.5. An odds ratio larger than 1 indicates a higher risk of SARS-CoV-2 infection, while an OR less than 1 indicates resistance to SARS-CoV-2. Meanwhile, the wide range of confidence intervals (Cis) indicated that the OR was based on the limited number of cases. Altogether, although the frequency of COVID-19 infection is higher with individuals with R219C variant, statistical confidence is pending by the lack of diagnosis records. Likewise, for the variants of K26R, the statistical significance is retained uncertain in the present study. The binding affinity between the K26R polymorphism in hACE2 and the S protein of SARS-CoV-2 was weak with mutated amino acids (ΔΔG value of 3.10); K26R of the hACE2-mutated group showed resistance to SARS-CoV-2 infections (OR 0.93 [0.38–2.27 of 0.95 CI]). Conversely, the R219C-mutated group showed susceptibility to SARS-CoV-2 infections with higher affinity for SARS-CoV-2 binding (ΔΔG value of -1.85). Due to the lack of a number of diagnostic tests for the A614S and Q86R variant groups, the OR values were absent.

**Table 3:** COVID-19 diagnostic test results by selected variants of hACE2.

Variant	ΔΔG (kcal/mol)	AF^b^	Odds ratio (0.95 of CI)	COVID-19 tests (N = 17,872)		Positive ratio	
				Minor allele (N)	Wild-type (WT)^a^	Minor allele (N)	WT
A614S	1.44	0.000404	-	1 (130)	100	0.0 (0)	0.06 (6)
R219C	-1.85	0.001067	1.5 (0.27–8.3)	6 (78)	600	0.33 (2)	0.24 (149)
Q86R	-1.32	7.70E-05	-	1(23)	100	0.0 (0)	0.04 (4)
K26R	3.10	0.006604	0.93 (0.38–2.27)	34 (2074)	3138	0.17 (6)	0.18 (584)

^a^ Selected wild-type subjects using propensity matching with minor allele group individuals who underwent COVID-19 diagnostic testing

^b^ Minor allele frequency

It should be noted that the positivity rates changed not only in the case of a contact residue (K26R) but also in other residues (R219C, Q86R and A614S). We also acknowledge that the magnitude of the change in the positivity rate was different depending on the amino acid. However, the UK Biobank data analysis suggested that the mutations in any locations in hACE2 can be associated with the infection rate with favorable consistency regarding binding affinity.

### Equilibrium molecular dynamics simulations

**Fig 2:**
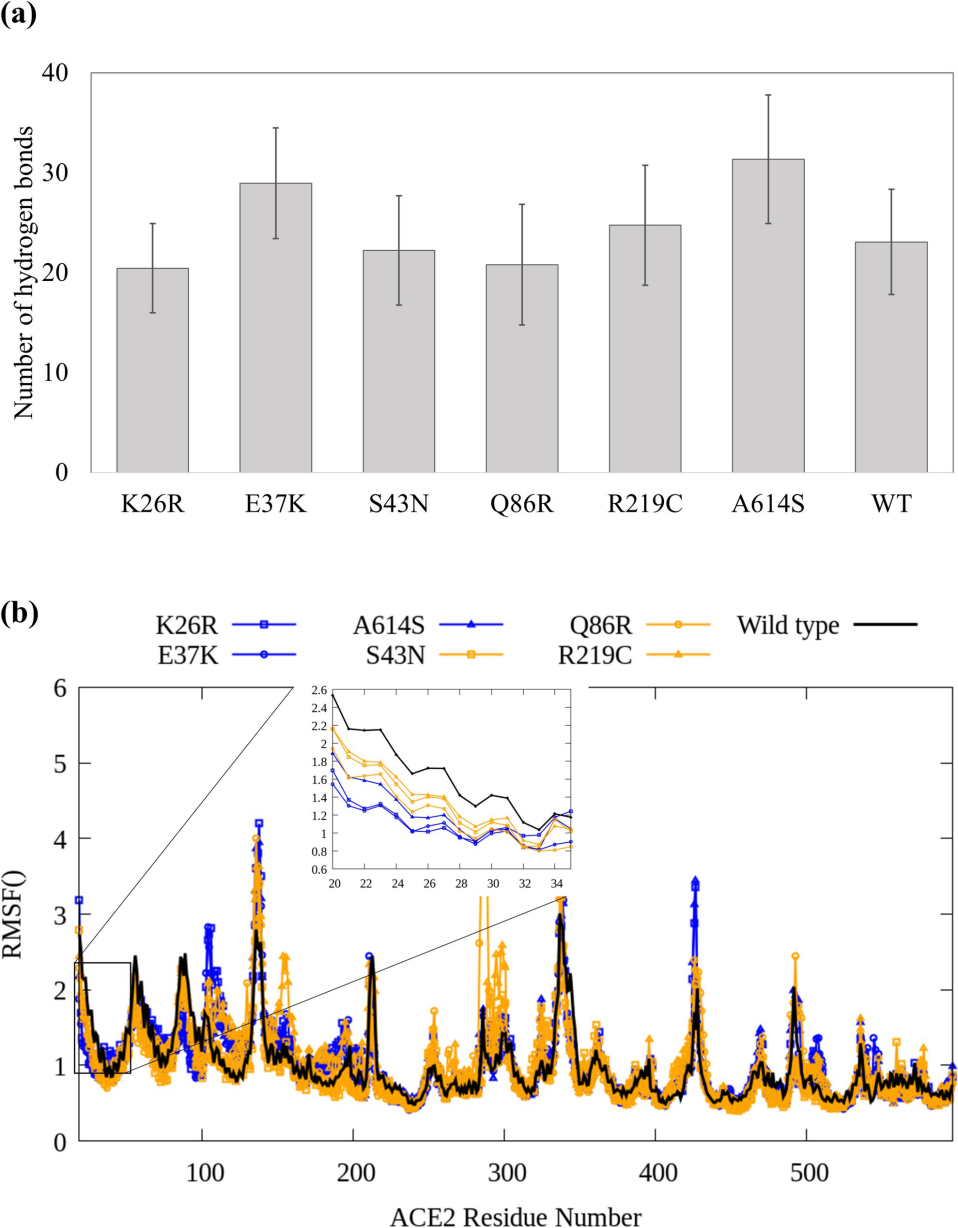
Analysis of hydrogen bonds and RMSF values. (a) Average number of hydrogen bonds from the 50 ns calculation. The error bar indicates the standard deviation. (b) Result of carbon alpha RMSF analysis.

For a deeper understanding of the molecular mechanisms by which mutated residues affect binding affinity, we analyzed the 100 ns equilibrium MD simulation trajectories. The RMSD showed equilibrium after 50 ns for the all system (**S1 Fig**). We first investigated hydrogen bonding and salt bridges between hACE2 and the S protein to characterize the contact interactions. **Fig 2A** depicts the average number of hydrogen bonds in the course of MD simulation. The wild-type structure showed an average of 23 hydrogen bonds. The mutations K26R, E37K and A614S, which showed positive ΔΔG changes, had 20.4, 28.9 and 31.3 hydrogen bonds, respectively. The mutations S43N, Q86R and R219C had 22.2, 20.8 and 24.7 hydrogen bonds, respectively. Except for E37K and A614S, most mutations yielded slightly different numbers of hydrogen bonds. In addition, the number of salt bridges did not change due to the mutations (**S1 Table**). Despite the fact that the mutations of selected residues induced changes in interfacial properties, we did not observe significant differences between mutations or find any consistency between free energy changes and the number of hydrogen bonds.

To investigate the effect of structural flexibility, we also calculated RMSF values (**Fig 2B**). The overall trend of the RMSF did not change significantly due to the mutations. However, the mutations led to RMSF value changes in flexible locations. It is notable that, in the case of the mutations that showed positive free energy changes (K26R, E37K and A614S), the RMSF values of contact residues (residues 20 to 34) yield smaller values compared to those of the WT group suggesting lesser structural flexibility (Fig 2(B) inset). Considering that the contact residues can directly affect the molecular binding between hACE2 and the S protein, the RMSF results indicated that the changes in structural flexibility due to mutation play an important role in binding affinity.

## Discussion

Various factors, such as race, sex and age, contribute to the SARS-CoV-2 infection rate. Not only medical aspects of SARS-CoV-2 risk but also social activity, such as that allowed by government policies, affect the incidence of COVID-19. Thus, due to the disease’s complexity, it is difficult to understand diverse COVID-19 infection rates depending on individuals. In this study, focusing on hACE2 variation, we investigated the effect of the genetic diversity of hACE2 on COVID-19 infection. We hypothesize that the infection rate is substantially related to mutations binding to the S protein of SARS-CoV-2. Our data analysis indicated that there is an associated pattern of co-occurrences between the infection rate and hACE2 variants from natural populations. Although there are many factors that influence the infection rate of the virus, including sociodemographic characteristics, such as access to hospitals, an analysis of the host’s genetic diversity corresponding to the entry of SARS-CoV-2 is presented for further study. As residues of hACE2 vary, the positivity rates of SARS-CoV-2 are altered. In addition, our data analysis and MD simulations indicated that not only the physicochemical properties of contact residues but also their structural flexibility have a putative role in binding.

Although we highlighted hACE2 as a binding partner of SARS-CoV-2, a number of studies presented different docking partners of the human host to SARS-CoV-2 infections, such as NRP1, L-SIGN(CLEC4M), DC-SIGN(CD209), and CD147(BSG) [27–30]. Therefore, the identification of germline variation in these genes can pave the way for further study. Using an identical method and UK Biobank data, we identified germline variants of these four partner genes of SARS-CoV-2. In Supplemental Data 1, we present the functional impact of germline variants based on the results of VEP analysis.

The originality of this study lies in addressing the impact of the genetic diversity of hACE2 on SARS-CoV-2 infections at the population scale. Owing to the host dependency for virus survival, the genome of SARS-CoV-2 suggests possible frequent recombinations and rapid evolution that has enabled the virus to adapt to the human host for transmission and pathogenesis. Due to an unprecedented number of SARS-CoV-2 infections, host-pathogen interactions have drastically increased, resulting in the appearance of variants of SARS-CoV-2, including Delta and Omicron [31]. Thus, for a prompt solution to the rapid evolution of SARS-CoV-2, an understanding of the existing variants in the host population against SARS-CoV-2 variants is indispensable. Because our framework is based on our supercomputing system (Nurion = 8,305 compute nodes and 563,740 central processing units (CPUs)), our approach would be scalable in the fight against the rapid evolution of the SARS-CoV-2 genome.

We acknowledge the limitations of this study. First, due to the limited number of positive cases, our result can hardly be interpreted as statistically valid. Nonetheless, this study presents clear evidence of which mutations of hACE2 affect binding affinity. Second, the effects of glycans attached to the surface of the S protein were not considered. It is widely known that the S protein protects itself from the immune system by glycans. Thus, glycosylation of the S protein may affect the binding free energy. However, due to limited structural information on glycosylation of the S protein, we did not explicitly model glycans. Despite its limitations, this study certainly adds to our understanding of the effect of hACE2 mutations on COVID-19 infection. Further analysis of the mutations depending on race, sex and age are needed and will provide insights for vaccine design. For example, the identification of hACE2 variants with higher levels of binding affinity with the spike protein of SARS-CoV-2 would allow us to detect an individual who already has the hACE2 variants with unfavorable docking properties for the spike protein. Thus, the identification of hACE2 variants among vaccinated persons contributes to estimating the protection efficacy of the designed vaccine. The identification of favorable bindings between hACE2 variants and the spike protein also catalyzes the design of strong antibodies for the spike protein of SARS-CoV-2. In addition, the identification of hACE2 variant frequency in a population also contributes to establishing a strategy to combat COVID-19.

## Method

### Analysis of genetic diversity and health related outcomes

To investigate the impact of hACE2 variation on its interaction with the S protein of SARS-CoV-2, we analyzed UK Biobank data covering over 500,000 individuals. Out of 502,543 participants, we utilized WES data of 200,643 individuals. We analyzed mean age, sex, degree of obesity, and Townsend deprivation index scores to present information on demographics, physical status, and background (i.e., social environment) of the UK Biobank participants. Using assigned diagnosis codes in inpatient cases, we also evaluated health-related outcomes between all individuals in the UK Biobank and the WES data subset of the UK Biobank. A total of 2,577,360 primary diagnoses from the inpatient records were analyzed. Out of those diagnosis records, 984,218 cases were from the hospital admission data of 200,643 WES-matched individuals. The UK Biobank Hospital Episode Statistics (HES) database contains inpatient records of all participants in the UK Biobank, including *International Classification of Diseases*, *Tenth Revision* (ICD-10) diagnosis codes, collected from external providers, such as hospitals in Scotland, England and Wales. The UK Biobank population of 500,000 participants comprises 89% of participants recruited in England, 7% recruited in Scotland, and 4% recruited in Wales. The HES records of the UK Biobank are collected longitudinally and retrospectively, covering inpatient records since 1996.

Using the Ensembl Variant Effect Predictor (VEP) [24], we assessed the functional impact of certain genetic variants of the hACE2 gene in each individual. With rule-based approaches, the VEP presents the consequences of each genetic variant, including synonymous, nonsense-mediated decay (NMD), and nonsynonymous variants (i.e., missense variants). Details are presented in **S1 File**. The minor allele frequency (AF) of those identified variants is also presented as a result of the VEP.

### Molecular dynamics simulation and free energy calculation

The crystal structure of the complex formed of hACE2 and the S protein of SARS-CoV-2 was obtained from the RCSB Protein Data Bank (PDB ID: 6M0J) [32]. Molecular dynamics simulations were performed using NAMD software [33]. The simulation systems with the complex of mutated hACE2 and the spike protein immersed in explicit water models were constructed with VMD and equilibrated with NAMD. The all-atom CHARMM36 force field and TIPT3P water model with rigid bonds were used throughout the MD simulation [34,35]. A constant temperature of 300 K and 1 atm of constant pressure were maintained with Langevin dynamics and the hybrid Nosé–Hoover–Langevin piston method on a flexible periodic cell, respectively [36–39]. The particle mesh Ewald method with a grid size of 1 Å was used for long-range electrostatics [40], and van der Waals interactions were treated with a switching distance of 10 Å and a smooth cutoff distance of 12 Å.

For alchemical free energy changes, we used the free energy perturbation (FEP) calculation method implemented in NAMD [41]. The alchemical transformation was carried out in forward and backward transitions with λ windows of 0.05 intervals. A softcore potential was applied in FEP. For one transition (i.e., λ was from 0.0 to 1.0), the FEP calculation was carried out using 4 windows. Each window was simulated for 1.1 ns, with the first 1 ns considered equilibration. Data collection was carried out every 0.2 ps for FEP analysis. Hence, a total simulation time of 8.8 ns for each FEP calculation was performed. We verified that the results from FEP runs of a total duration of 8–10 ns were closely reproducible. The starting complex FEP structures of the hACE2-S protein complex were modeled with the VMD Mutator plugin.

For equilibrium MD simulations of mutation, we constructed the initial protein structures with SWISS-MODEL [42]. We ran equilibrium MD simulations for 100 ns, and the last 50 ns simulation trajectories were used for analysis. Analyses of hydrogen bonds, the RMSF and the RMSD were conducted using plug-in modules included in VMD.

## Supporting information

S1 File The functional impact of given genetic variations of selected genes using Ensemble Variant Effect Predictor. (XLSX)

S1 Table The analysis result of salt bridges and hydrogen bonds. (PDF)

S1 Fig RMSD values of hACE2-S protein complex. (PNG)
