# Supplementary material for: Analysis of the docking property of host variants of hACE2 for SARS-CoV-2 in a large cohort
Source: PLoS Comput Biol. 2022 Jul 11;18(7):e1009834. doi: 10.1371/journal.pcbi.1009834 (PMC9302733; doi:10.1371/journal.pcbi.1009834)
Supplement: S1 Table — (PDF) [file pcbi.1009834.s002.pdf]

Table S1 The analysis result of salt bridges and hydrogen bonds

| | **Number of salt bridges** | **Average number of hydrogen bonds** |
|---|---|---|
| **Crystal structure** | 1 | |
| **Wild-type** | 1 | 23.07±5.26 |
| **K26R** | 1 | 20.45±4.45 |
| **E37K** | 1 | 28.95±5.53 |
| **S43N** | 1 | 22.23±5.47 |
| **Q86R** | 1 | 20.80±6.03 |
| **R219C** | 1 | 24.75±5.99 |
| **A614S** | 1 | 31.35±6.43 |
