# Supplementary figures and images for: Analysis of the docking property of host variants of hACE2 for SARS-CoV-2 in a large cohort
Source: PLoS Comput Biol. 2022 Jul 11;18(7):e1009834. doi: 10.1371/journal.pcbi.1009834 (PMC9302733; doi:10.1371/journal.pcbi.1009834)

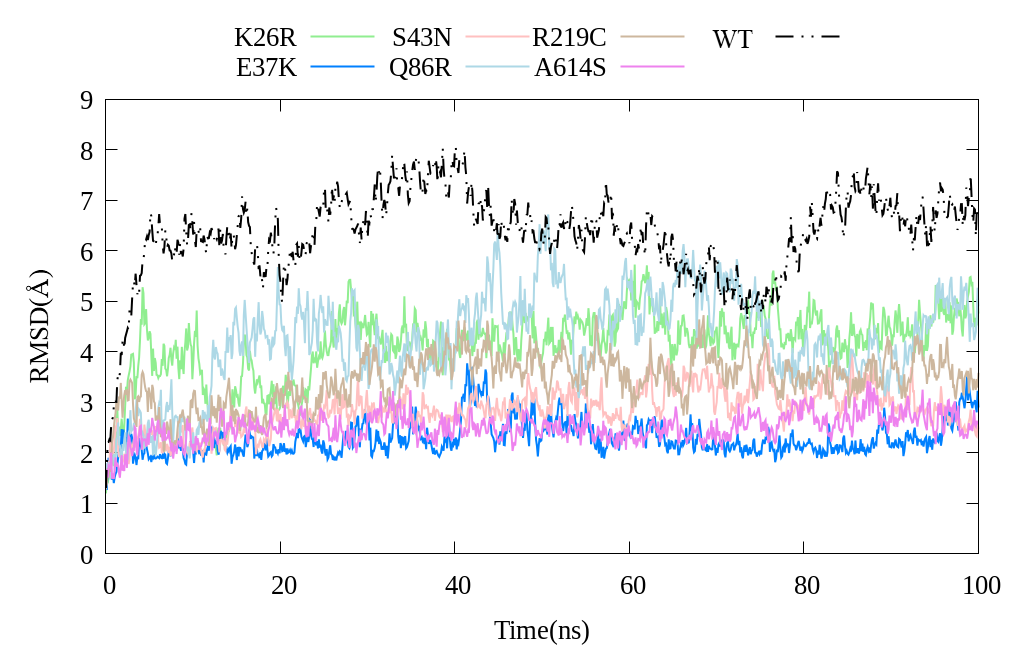

Supplement: S1 Fig — (PNG) [file pcbi.1009834.s003.png]
